# Neuro-Behçet’s Syndrome: A Case Report

**DOI:** 10.7759/cureus.84421

**Published:** 2025-05-19

**Authors:** David I LeRoy, Elaine Ognjanovski, Silvia Menkes, Kristen Kenny, Cassie Konja

**Affiliations:** 1 Internal Medicine, Henry Ford Health System - Warren, Warren, USA; 2 Neurology, Henry Ford Health System - Warren, Warren, USA

**Keywords:** behçet's syndrome, infliximab, neuro-behçet's syndrome, seizure, status epilepticus

## Abstract

Behçet's syndrome is a systemic vasculitis characterized by recurrent oral and genital ulcers, uveitis, and often involvement of multiple organ systems. Neurological involvement is a rare complication of Behçet's syndrome and is associated with significant morbidity and mortality. Neuro-Behçet's syndrome (NBS) is a combination of neurologic manifestations in a patient with Behçet's syndrome, including movement disorders, myelopathic syndrome, intracranial hypertension, and a multiple sclerosis-like presentation. Diagnosis of NBS can be challenging due to the wide range of clinical presentations. Although previous research has aided in advancing the understanding of the pathophysiology and treatment of systemic Behçet's syndrome, there is a lack of randomized controlled studies involving the treatment of NBS. This case report describes a patient with NBS who experienced a complex hospital course, highlighting the need for a multidisciplinary team approach to address various complications.

## Introduction

Behçet's syndrome is a chronic, multisystemic, relapsing vasculitis affecting both small and large blood vessels. Behçet's syndrome is classically characterized by recurrent oral aphthae, genital ulcers, arthritis, uveitis, and thrombophlebitis. This syndrome has also been described as having involvement in the gastrointestinal and nervous systems [1]. Neurological involvement in Behçet's syndrome is a rare manifestation described in 5-10% of patients [2]. It is one of the most severe manifestations of Behçet's syndrome and has been associated with mortality of ~10% [3,4]. The diagnosis of neurological involvement of Behçet's syndrome remains challenging due to the absence of a specific diagnostic test [5].

Neuro-Behçet's syndrome (NBS) can be categorized into central nervous system (CNS) and peripheral nervous system (PNS) manifestations, with CNS involvement being further divided into parenchymal and nonparenchymal [5]. CNS parenchymal involvement is the most common subtype of NBS, affecting approximately 75-80% of NBS cases [6]. Symptoms of parenchymal involvement may include hemiparesis, dysarthria, ataxia, cranial neuropathies, and/or headaches [4]. It can be further classified into acute progressive vs. chronic progressive parenchymal NBS. Acute flare-ups of NBS are often treated with high-dose glucocorticoid steroids followed by slow tapers. Chronic progressive parenchymal NBS has a more complex course and may cause symptoms refractory to corticosteroids and immunosuppressive medications [7]. TNF (tissue necrosis factor) inhibitors can be utilized for severe cases, with TNF-alpha inhibitors as a possible alternative for refractory cases [6]. Nonparenchymal involvement may include pseudotumor cerebri, cerebral venous thrombosis, and, rarely, stroke [8].

This case was presented at the 2025 Michigan Chapter American College of Physicians and Society of Hospital Medicine Michigan Chapter Resident and Medical Student Day on May 2, 2025.

## Case presentation

**Figure 1:**
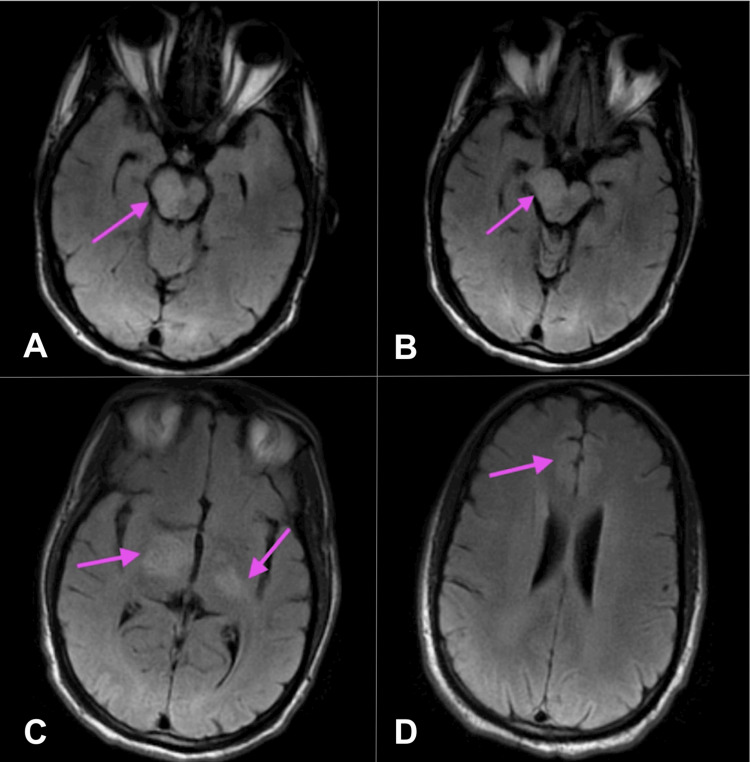
T2 FLAIR MRI brain without contrast (A-D) Differing axial view depths of the brain. MRI brain without contrast (T2 FLAIR) showing hyperintense area extending from the mid-right pons through the midbrain, thalamus, and into the posterior limb of the right internal capsule, concerning for demyelination. Arrows highlight the areas of concern. FLAIR: fluid-attenuated inversion recovery; MRI: magnetic resonance imaging

A 41-year-old African American male patient with a past medical history of Behçet's syndrome (on infliximab infusions as an outpatient) presented to the emergency department with a two-day history of slurred speech and gait difficulties. On examination, his NIH Stroke Scale score was 5 out of 42, indicating a mild stroke, notable for partial facial paralysis, left upper and lower extremity drift, and mild to moderate slurred speech. A CT head without contrast obtained on admission revealed no evidence of ischemic or hemorrhagic stroke. CTA (computed tomography angiography) of the head and neck revealed no acute intracranial abnormalities or evidence of high-grade stenosis or occlusion. A follow-up MRI of the brain without contrast demonstrated a T2 FLAIR (fluid-attenuated inversion recovery) hyperintensity extending from the mid-right pons through the midbrain and thalamus into the posterior limb of the right internal capsule, as seen in Figure 1.

At this time, the patient reported compliance with outpatient infliximab, which raised concern for acute disseminated encephalomyelitis as a potential complication of infliximab therapy. The patient was initiated on intravenous (IV) methylprednisolone at 500 mg every 12 hours for a total of six doses, followed by continued pulse-dose steroids. Rheumatology was consulted during this time and recommended outpatient discontinuation of infliximab without additional inpatient workup. The patient completed this course of steroids with clinical improvement, and plans were made to discharge to inpatient rehabilitation, which was delayed due to insurance.

**Figure 2:**
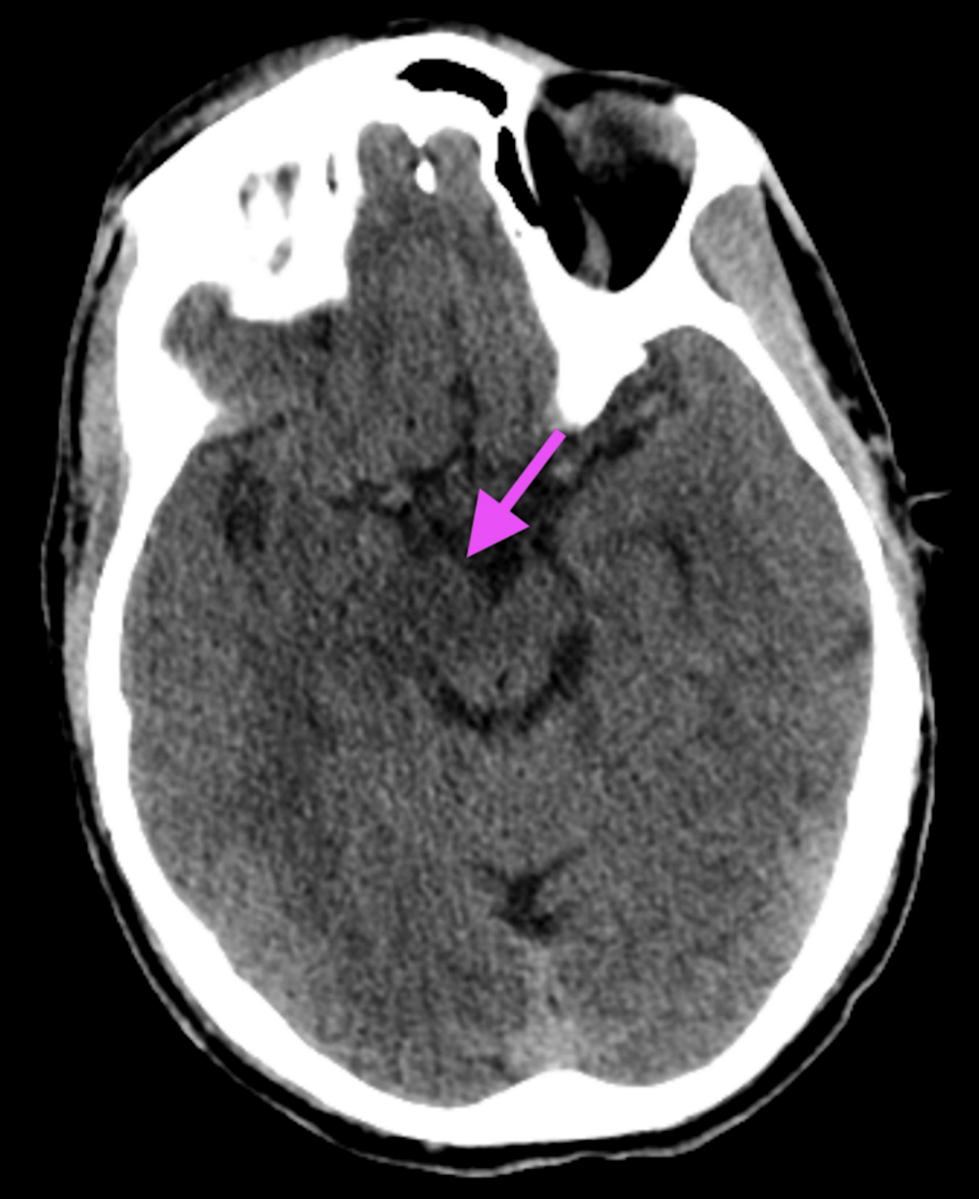
CT head A CT scan of the head shows an asymmetrical edema in the right cerebral peduncle (arrow), which extends into the upper pons.

While awaiting insurance authorization, a rapid response was called for status epilepticus. The patient was treated with 2 mg of lorazepam twice, followed by 1 g of levetiracetam for tonic-clonic seizures, which persisted for 10 minutes despite treatment. He was subsequently administered an additional 2 mg of IV lorazepam. He underwent a repeat CT head, which demonstrated an asymmetrically edematous right cerebral peduncle extending into the upper pons, as seen in Figure 2.

**Figure 3:**
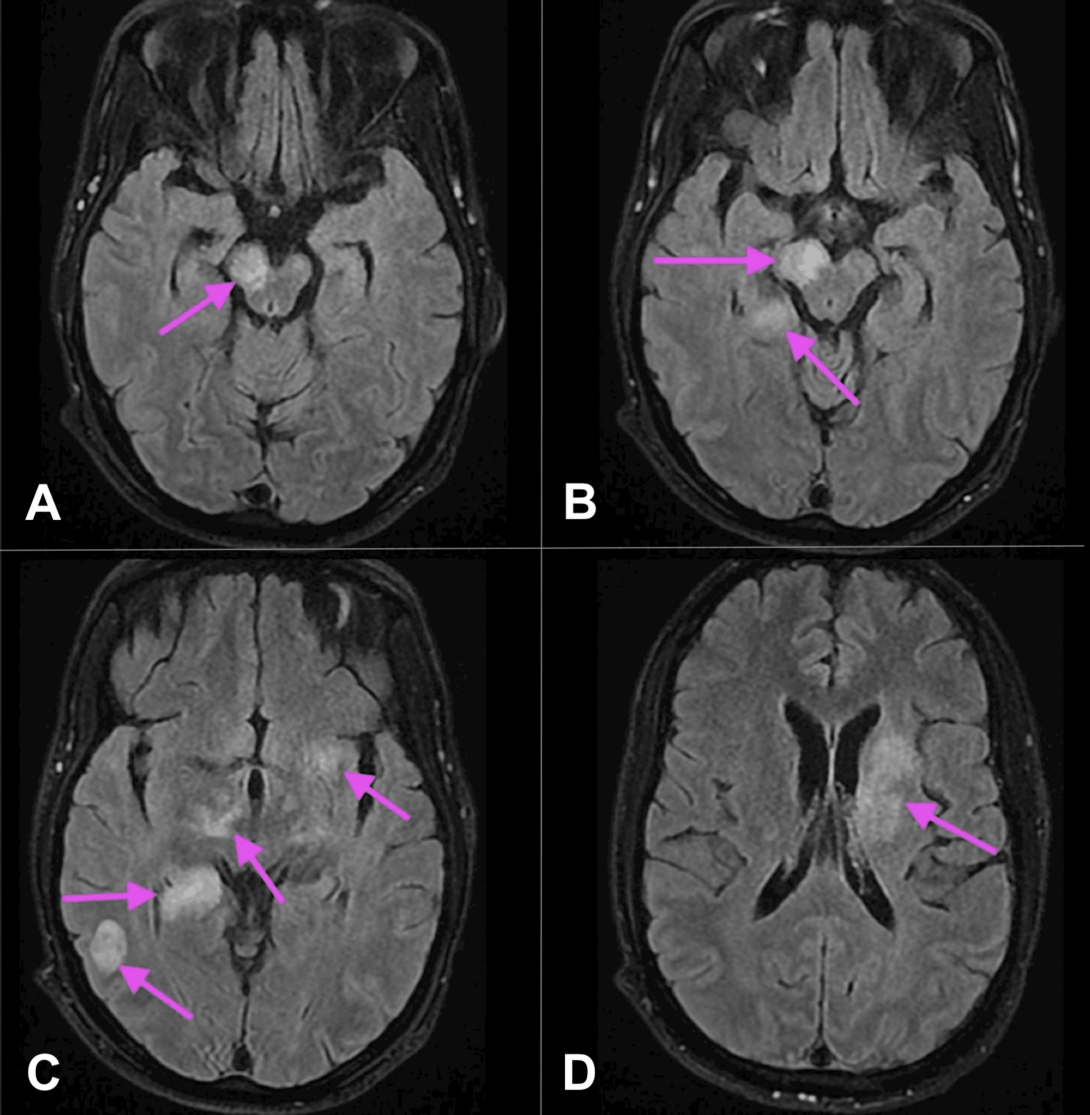
T2 FLAIR MRI brain with and without contrast (A-D) Differing axial view depths of the brain. A repeat MRI of the brain using T2 FLAIR with and without contrast shows active demyelination in the left dorsal medulla, the posterior right hippocampus, the right temporo-occipital subcortical white matter, and both the left corona radiata and basal ganglia. Large chronic demyelinating lesions are noted within the right midbrain, cerebral peduncle, and caudal thalamus. Small chronic demyelinating lesions are seen within the left thalamus and internal capsule. Arrows highlight the multiple areas of demyelination. FLAIR: fluid-attenuated inversion recovery; MRI: magnetic resonance imaging

At this point, the patient had been off steroids for three days. He was transferred to the Step-Down Unit; however, the patient was unable to protect his airway and was subsequently intubated and transferred to the ICU. While in the ICU, a repeat MRI of the brain was ordered, demonstrating an increase in active demyelination with hyperintensities within the left dorsal medulla, posterior right hippocampus, right temporo-occipital subcortical white matter, and left corona radiata and basal ganglia, as seen in Figure 3.

Additionally, the MRI demonstrated large chronic demyelinating lesions within the right midbrain, cerebral peduncle, and caudal thalamus. It also demonstrated a small chronic demyelinating lesion within the left thalamus and internal capsule. During this time in the ICU, the patient underwent a lumbar puncture with cerebrospinal fluid (CSF) analysis showing 7 mm^3^ lymphocyte-predominant WBCs, 46 mg/dL protein, and 64 mg/dL glucose. These studies were negative for bacterial or fungal growth, as well as for possible viral etiology. After a total of two days of being intubated, the patient was extubated and was then transferred to the general medical floor.

Due to clinical and radiographic evidence of decompensation, the case was further discussed with rheumatology. They discovered that he was initially diagnosed in 2016 after developing recurrent oral and genital lesions and has been following up with an outpatient rheumatologist since then, with later hospitalization for focal neurologic deficits. MRI revealed CNS involvement. The patient was initially started on mycophenolate mofetil, azathioprine, and prednisone. Due to syndrome progression, his treatment was transitioned to infliximab infusions every six weeks, which he has been receiving for the past few years. However, he discontinued this regimen three months prior to the current presentation in preparation for an upcoming hernia repair surgery. With the new information revealing that the patient had been off infliximab for approximately five months, it was theorized that the lapse in immunosuppressive therapy led to the recrudescence of the CNS manifestations of his Behçet's syndrome.

Given the patient's significant improvement from the previous course of IV steroids, he was started on methylprednisolone 1 g IV for two days with plans to transition him to oral prednisone at 30 mg twice daily with a prolonged taper. The patient showed improvement in weakness on this regimen and was discharged to inpatient rehabilitation. There, he continued to improve on oral prednisone, with plans to maintain the dose at 30 mg twice daily for one month, followed by a taper of 10 mg weekly until reaching 30 mg daily. The patient was discharged home and instructed to follow up with his outpatient rheumatologist.

## Discussion

Neurological involvement is a rare manifestation of Behçet's syndrome. Classically, non-neurologic symptoms such as intermittent oral and genital aphthous lesions precede neurological involvement, which generally occurs approximately five to six years following initial symptom onset [5,9]. NBS symptoms depend on the area of involvement. Areas most commonly affected are within the CNS parenchyma, primarily the brainstem and basal ganglia [5]. Additionally, 40% of patients with NBS will have relapses of their symptoms, with 50% developing moderate to severe disability and cognitive impairment [4]. MRI is considered the gold standard diagnostic modality, as it effectively distinguishes between acute lesions and acute infarcts [4,8]. Further studies have shown that CSF analysis may also be utilized in the diagnosis of parenchymal NBS, as 70-80% have altered CSF analysis with absent oligoclonal bands, elevated proteins, and elevated cell counts [4]. However, nervous tissue biopsy and pathergy testing are generally not recommended in the diagnosis of NBS [4].

Seizures have been described in various autoimmune disorders, including NBS. NBS has been associated with parietal seizures, generalized tonic-clonic seizures, myoclonic jerks, epilepsia partialis continua, and status epilepticus [2]. Currently, seizures in NBS are thought to be mainly due to increased intracranial pressure rather than inflammatory changes within the nervous system [2]. Status epilepticus in patients with NBS has rarely been described in studies. A recent study observed seizures in 16.7% of patients with NBS. These patients were treated with various antiepileptic drugs, including carbamazepine and levetiracetam [2]. Our patient was given 1 g of IV levetiracetam for a tonic-clonic seizure; however, his seizure required 6 mg of IV lorazepam to break. While a rare complication of NBS, seizures are an important, deadly complication to monitor for when treating patients with NBS.

Treatment for NBS primarily relies on experienced neurologists and rheumatologists to manage this syndrome, as there is a lack of randomized controlled trials (RCTs) to confirm the efficacy of standardized treatments. Most neurologists will treat acute flare-ups and relapses with IV methylprednisolone 1 g followed by a slow taper of oral steroids [8]. Treatment of NBS can also utilize azathioprine, a purine analogue, for immunosuppression in Behçet's syndrome. Azathioprine is used as a first line for ocular involvement and can be used to treat deep vein thrombosis, enteric, and joint manifestations; however, no RCTs show true efficacy in NBS [10].

Mycophenolate mofetil, a prodrug of mycophenolic acid, is an inhibitor of inosine-5’-monophosphate dehydrogenase, which can be used to treat NBS. There has been a case series of four patients reporting efficacy of mycophenolate mofetil 2 g/day without relapse within three to seven years [10]. Despite efficacious results, the generalization of this study is limited due to its low power. Recent advancements in biologic medications have allowed monoclonal antibodies such as infliximab to be successfully utilized in the management of Behçet's syndrome. Infliximab, a TNF-alpha inhibitor, has been widely used in various manifestations, including ocular, enteric, neuro, vasculitis, and arthritis [10,11]. Although infliximab is typically well tolerated, there have been reported cases of infections, congestive heart failure, hemocytopenia, and T cell lymphoma, as well as a growing number of cases of demyelination [12]. Current French recommendations for the management of NBS include systemic corticosteroid therapy at 1 mg/kg/day with or without an IV bolus of 500 mg/day for three days in cases of parenchymal disease [13]. For moderate disease, azathioprine (2 mg/kg/day) or methotrexate (0.3 mg/kg/week) may be used, while serious disease may require cyclophosphamide IV or an anti-TNF-alpha such as infliximab [13].

In this case, imaging originally suggested that demyelination was secondary to infliximab use. However, upon further investigation, the patient was found to have an exacerbation of his NBS with worsening lesions as a result of infliximab discontinuation. He was treated with 1 g IV methylprednisolone followed by a prolonged oral steroid taper starting at 30 mg twice daily, which helped resolve his acute flare. While other cases have utilized this treatment for acute exacerbations, further research through RCTs is needed to determine a standardized treatment protocol. This syndrome carries an increased mortality risk, necessitating an optimal treatment protocol for both standard NBS and more treatment-resistant NBS. Moreover, further investigation is needed to evaluate the risk of epileptic activity and standardize the treatment for epilepsy in this population. Studies are currently divided on whether patients benefit from long-term antiepileptic therapy [2]. Areas that require further study include determining why patients with NBS carry an increased risk of developing seizures and which antiepileptic drug regimen is most efficacious for this patient population.

## Conclusions

NBS is a rare manifestation of Behçet's syndrome that clinicians should be aware of, as it is associated with an increased mortality risk. Early differentiation between strokes and NBS is highly important, as NBS more commonly affects the CNS and can present similarly to a stroke. Epilepsy is rarely seen in patients with NBS. Consequently, there is a lack of research on standardized treatment for epilepsy in patients with NBS. Numerous medications used as treatment for NBS lack RCTs. With biologic agents, advancements in therapy bring new hope to the treatment of this syndrome.

## References

[1] Caruso P, Moretti R (2018). Focus on neuro-Behçet's disease: a review. Neurol India.

[2] Kutlu G, Semercioglu S, Ucler S, Erdal A, Inan LE (2015). Epileptic seizures in neuro-Behcet disease: why some patients develop seizure and others not?. Seizure.

[3] Herrero-Morant A, Martín-Varillas JL, Castañeda S (2022). Biologic therapy in refractory neurobehçet's disease: a multicentre study of 41 patients and literature review. Rheumatology (Oxford).

[4] Yazici Y, Hatemi G, Bodaghi B, Cheon JH, Suzuki N, Ambrose N, Yazici H (2021). Behçet syndrome. Nat Rev Dis Primers.

[5] Borhani-Haghighi A, Kardeh B, Banerjee S, Yadollahikhales G, Safari A, Sahraian MA, Shapiro L (2020). Neuro-Behcet's disease: an update on diagnosis, differential diagnoses, and treatment. Mult Scler Relat Disord.

[6] Karadag O, Bolek EC (2020). Management of Behcet's syndrome. Rheumatology (Oxford).

[7] Hirohata S, Kikuchi H, Sawada T (2020). Recommendations for the management of neuro-Behçet's disease by the Japanese National Research Committee for Behçet's Disease. Intern Med.

[8] Kalra S, Silman A, Akman-Demir G (2014). Diagnosis and management of Neuro-Behçet's disease: international consensus recommendations. J Neurol.

[9] Peine B, Figueroa C, Robinette N (2022). Neuro-Behcet's syndrome: case report and literature review. Radiol Case Rep.

[10] Rodríguez-Carrio J, Nucera V, Masala IF, Atzeni F (2021). Behçet disease: from pathogenesis to novel therapeutic options. Pharmacol Res.

[11] Barešić M, Reihl M, Habek M, Vukojević N, Anić B (2018). Improvement of neurological and ocular symptoms of Behçet's disease after the introduction of infliximab. Rheumatol Int.

[12] Kemanetzoglou E, Andreadou E (2017). CNS demyelination with TNF-α blockers. Curr Neurol Neurosci Rep.

[13] Kone-Paut I, Barete S, Bodaghi B (2021). French recommendations for the management of Behçet's disease. Orphanet J Rare Dis.

